# Cryo‐EM structures of amyloid beta and tau filaments in down syndrome

**DOI:** 10.1002/alz.094953

**Published:** 2025-01-09

**Authors:** Anllely Fernandez, Md Rejaul Hoq, Grace Hallinan, Daoyi Li, Sakshibeedu R. Bharath, Frank S. Vago, Kadir A. Ozcan, Kathy L Newell, Holly J. Garringer, Wen Jiang, Bernardino Ghetti, Ruben Vidal

**Affiliations:** ^1^ Indiana University, INDIANAPOLIS, IN USA; ^2^ Purdue University, West Lafayette, IN USA

## Abstract

**Background:**

Down syndrome (DS) is the most common and best‐known chromosomal disorder in humans and the most frequent cause of intellectual disability of genetic origin, affecting about 6 million people worldwide. Individuals with DS may develop Alzheimer disease (AD) by age 55‐60 years, and sometimes as young as 40 years due to the triplication of the amyloid β precursor protein (AβPP) gene, which is located on chromosome 21. The AD neuropathological phenotype in DS includes amyloid‐β (Aβ) deposition (parenchymal and vascular) and neurofibrillary tangles comprised of tau protein. Aβ peptide species ending at 42 (Aβ_42_) are the main component of senile plaques and diffuse deposits in AD and AD in DS, while Aβ peptides ending at position 40 (Aβ_40_) are the predominant Aβ peptides found in both leptomeningeal and cortical vessels. Whether there is a difference in the structures of Aβ and tau filaments between AD and AD in DS, is unknown.

**Method:**

We used cryo‐electron microscopy (cryo‐EM) to study the structure of Aβ and tau filaments extracted from the brains of two individuals with DS. Both individuals had been clinically diagnosed with AD, which was neuropathologically confirmed.

**Result:**

We found two types of Aβ_42_ filaments (I and II) identical to those found in sporadic and familiar AD and two novel Aβ_40_ filaments (type IIIa and IIIb) that differ from those previously reported in AD. Tau filaments (paired helical filaments, PHFs, and straight filaments, SFs), were identical to those from AD and related diseases.

**Conclusion:**

This cryo‐EM study emphasizes the similarities and differences between amyloid filaments in AD and AD in DS. The relevance of the structural differences between Aβ_40_ filaments in cerebral amyloid angiopathy in AD and DS is unknown. Further research is needed to determine whether type IIIa and IIIb Aβ_40_ filaments are unique to DS. Tau filaments (PHFs and SFs) were identical to those in AD, supporting the notion of a common mechanism through which amyloids trigger aggregation of tau. This knowledge is crucial for understanding AD in individuals with DS and assessing whether adults with DS could be included in AD clinical trials.

